# Predicting the Future: Parental Progeny Investment in Response to Environmental Stress Cues

**DOI:** 10.3389/fcell.2019.00115

**Published:** 2019-06-19

**Authors:** Leah Gulyas, Jennifer R. Powell

**Affiliations:** Department of Biology, Gettysburg College, Gettysburg, PA, United States

**Keywords:** terminal investment, maternal effects, epigenetics, stress response, progeny fitness

## Abstract

Environmental stressors can severely limit the ability of an organism to reproduce as lifespan is decreased and resources are shifted away from reproduction to survival. Although this is often detrimental to the organism’s reproductive fitness, certain other reproductive stress responses may mitigate this effect by increasing the likelihood of progeny survival in the F1 and subsequent generations. Here we review three means by which these progeny may be conferred a competitive edge as a result of stress encountered in the parental generation: heritable epigenetic modifications to nucleotides and histones, simple maternal investments of cytosolic components, and the partially overlapping phenomenon of terminal investment, which can entail extreme parental investment strategies in either cytosolic components or gamete production. We examine instances of these categories and their ability to subsequently impact offspring fitness and reproduction. Ultimately, without impacting nucleotide sequence, these more labile alterations may shape development, evolution, ecology and even human health, necessitating further understanding and research into the specific mechanisms by which environmental stressors are sensed and elicit a corresponding response in the parental germline.

## Introduction

Under a traditional Darwinian view of evolution, variation in organismal fitness from generation to generation was thought to be almost exclusively due to germline nucleotide mutations, and non-genetic heritability was discounted. However, our current understanding of progeny investment and fitness reveals a far more nuanced, complex model, where environmental cues present in one generation can have lasting transgenerational impacts on offspring physiology and survival without impacting the DNA sequence. Various forms of environmental stimuli such as nutrient availability, pathogen exposure, and other xenobiotic factors may induce parental responses that attempt to better prepare offspring for prevailing environmental conditions. As a form of stress response, this directly opposes traditional views on life-history tradeoffs (Duffield et al., 2017), in which stressed organisms limit energy expenditure on reproduction to allocate more toward organismal defense and repair, a “survive today, reproduce tomorrow” strategy. Conversely, targeted reproductive investment and appropriate offspring provisioning in response to stress has the obvious benefit of maximizing offspring survival in harsh environments.

What cellular processes underlie these “reproductive stress responses,” manifesting in either increased gamete production or improved gamete/embryo quality? Here we consider the following categories of parental investment and their cellular bases: epigenetic modification, simple maternal effects, and terminal investment. Each of these forms of investment can have a substantial impact on progeny fitness by different means. On the level of DNA architecture, epigenetics play an important role in offspring survival through the inheritance of histone or nucleotide modifications that alter chromatin structure, influencing gene expression. Conversely, what we term “simple maternal effects” present more holistic changes to the cytosolic composition of gametes and offspring as a result of macromolecular loading from the parental germline. This is distinguished from the similar and partially overlapping phenomenon of terminal investment, which we will define as meeting the following criteria: (i) the current internal and external environment must elicit a high likelihood of mortality for the organism and (ii) the investment must represent a substantial reallocation of resources such that a subset of offspring benefit, but there is a significant cost to the organism’s survival and chance of future reproduction. We examine these three categories of cellular parental investment in a broad range of taxa, including a brief look at their evolutionary basis and significance.

## Epigenetics and Chromatin Modifications

Epigenetic marks are chemical modifications to either nucleotides or histone tails that modulate gene expression by serving as a physical barrier to transcription factors or by altering chromatin condensation, changing the accessibility of genes (Swygert and Peterson, 2014). Within an organism’s lifespan, epigenetic effects cause durable phenotypic plasticity in response to stressors, but modifications may also be propagated transgenerationally (Heard and Martienssen, 2014; D’Urso and Brickner, 2017). Consequently, the epigenome can impact the transcriptome and fitness of progeny in a lineage, conveying environmental information across generations.

Numerous examples attest to the importance of the epigenome for offspring-environment compatibility. In humans, Sun et al. (2018) provide evidence that offspring conceived during seasonal cold temperatures are more likely to possess active brown adipose tissue needed for thermogenesis, which additionally correlates to lower body mass index. This correlation is mirrored in mice and coincides with cold-induced epimutations in murine sperm, suggesting that epigenetics play a role in preparing offspring for cold environments. Similar phenomena frequently occur in plants (Crisp et al., 2016). Exposure to salt stress, heat stress, and herbivory in the parental generation of some species promotes resilience in progeny when they are later exposed to the same or similar stressors (Whittle et al., 2009; Boyko et al., 2010b; Rasmann et al., 2012). In some cases, there is not a direct input–output relationship between the type of stress encountered and the acquired stress resistance (Slaughter et al., 2012; Kishimoto et al., 2017). This may suggest that some epigenetic response mechanisms, rather than being specific for a certain stressor, are a more general priming of multiple and overlapping stress response pathways.

Establishment and propagation of the epigenome is particularly remarkable given that many organisms undergo massive reprogramming during early embryonic development, eliminating many key epigenetic marks from parental chromatin. Most modified 5-methylcytosine bases in mammals revert to cytosine to enable differential methylation in cell lineages via the *de novo* methyltransferases DNMT3a and DNMT3b (Wossidlo et al., 2011; Hackett and Surani, 2013). However, current studies have demonstrated that methylation state information can be successfully transmitted transgenerationally. Mice exposed to an olfactory fear stimulus exhibit specific hypomethylation in an olfactory gene in both parental gametes prior to conception that remains present in gametes several generations after exposure, substantially impacting neuroanatomy (Dias and Ressler, 2014). Gillette et al. (2018) additionally show that endocrine-disrupting toxin exposure in mice causes differential methylation at promoter CpG islands that remains stable at least three generations thereafter.

The histone code inherited from parental gametes is also broadly altered during early embryogenesis in some organisms; yet histone modifications induced by stress can be stably inherited (Morgan et al., 2005). Low levels of heat, osmotic, or heavy metal stress in the nematode *Caenorhabditis elegans* potentiate an increased resistance to oxidative stress in both the F1 and F2 generations. These transgenerational effects result from the activity of the histone H3 lysine 4 trimethylase complex in the germline, which is also required for transgenerational mark maintenance (Kishimoto et al., 2017). In parthenogenetic *Artemia*, low levels of heat stress likewise increase both heat and pathogen resistance for multiple generations by altering histone H3 and H4 global methylation and acetylation patterns (Norouzitallab et al., 2014). Altogether, the persistence of epigenetic marks from parent to offspring indicates that some modifications must survive the reset or be subsequently reestablished. It is important to note that epigenetic processes are not necessarily conserved across species. Studies are also impeded by several methodological challenges, such as the difficulty of assessing properly controlled samples in the correct post-stimulus generation (Skvortsova et al., 2018).

The mechanistic link between stress perception and epigenetic modification is not well understood. Promising candidates for mediators acting at the interface of environmental information and epimutation are non-coding RNAs. For example, small RNAs are implicated as essential elements in stress response pathways in plants such as *Arabidopsis thaliana*. Here, heritable epigenetic changes in response to environmental stimuli are dependent on proteins responsible for small RNA biogenesis, leading to the proposal that methylation changes are directed, at least in part, by stress-associated non-coding RNAs (Matzke et al., 2007; Boyko et al., 2010a). In mice, a complex endocrine signaling pathway that causes vesicular trafficking of miRNA from the epididymis to sperm is thought to contribute to germ cell reprogramming (Chan et al., 2018). Though these examples highlight the importance of non-coding RNAs, they also underscore the enormous variation in epigenetic mechanisms and the difficulty of determining whether conservation exists across organisms.

The evolutionary occurrence of such mechanisms is more intuitive; epigenetic modifications in gametes, as opposed to more costly cytosolic investment, is a relatively inexpensive and persistent means of provisioning progeny for a predictable future. In terms of fitness, the epigenome introduces the possibility of directly selecting for or against a phenotype, but not an associated genotype. Adaptations could thus arise very quickly in populations where multiple individuals simultaneously express different beneficial epigenetic marks, increasing “mutability,” (or rather changes in phenotypic diversity) in a way that does not alter DNA sequence (Jablonka and Raz, 2009). Comprehensive reviews of epigenomic evolution investigate these ideas more thoroughly (Jablonka and Lamb, 2005; Jablonka and Raz, 2009).

## Simple Maternal Effects

Variable environmental conditions and stressors may produce a situation where changes to the cytosolic makeup of gametes and embryos confer a fitness advantage to the F1 generation. These simple parental investments are most obvious in the maternal line as a result of the increased cytosolic investment in the maternal versus paternal gametes of most organisms. In our discussion of simple maternal effects, we will consider examples where the severity of the stressor and the strength of the investment do not entail a considerable risk of mortality.

In many cases, simple maternal effects are the inclusion of specific biomolecules tailored to the prevailing conditions. For example, *C. elegans* exposed to hyperosmotic environments during adulthood upregulate embryonic investment of the cryoprotectant glycerol at the expense of energy-rich glycogen (Frazier and Roth, 2009). Transgenerational immune priming similarly relies on the passage of small molecules to offspring. Several studies in insect species have documented maternal trafficking of inactive pathogen fragments to eggs, where they act as immune elicitors (Knorr et al., 2015; Salmela et al., 2015). In honey bees (*Apis mellifera*), this is accomplished using yolk proteins to bind, shuttle, and incorporate fragments into eggs (Salmela et al., 2015). Immune priming in invertebrates plays an important role in offspring survival by regulating the expression of immune-related genes and promoting faster infection clearance and lower pathogen loads when those offspring are challenged with a pathogen (Freitak et al., 2014; Rosengaus et al., 2017). Vertebrates comparably transmit immunity to developing embryos via the passage of antibodies through the mammalian placenta or avian egg yolk (Hasselquist and Nilsson, 2009).

Maternal effects are also commonly mediated by mRNA deposition, which substantially impacts offspring survival during stress. In *Drosophila*, one of the most abundant transcripts incorporated into oocytes codes for the small heat shock protein, HSP23; when overexpressed in maternal ovaries, increased *hsp23* transcript loading mitigates larval impairment following embryonic heat shock (Lockwood et al., 2017). Though upregulation of *hsp23* mRNA deposition has not yet been shown to occur in heat-shocked mothers, mRNA deposition plasticity appears in other examples in flies, particularly in relation to nutritional status. Crofton et al. (2018) demonstrate that mothers that were nutrient-limited as larva produce embryos depleted of certain mRNA transcripts involved in translation and enriched for those involved in biomolecule localization and transport. This may limit the energetically expensive translation process while mitigating the protein and mRNA mislocalization occurring in inadequately nourished oocytes.

Other studies of nutrient deprivation display more general dimensional and volumetric changes to offspring. *C. elegans* faced with dietary restriction display an insulin-dependent increase in both offspring size and resistance to early larval starvation (Hibshman et al., 2016). Weight plasticity has also been documented in *Drosophila*, where poorly fed larvae mature to produce offspring with 3–6% greater weight, despite attaining lower adult weight themselves. These offspring further show increased developmental rates when malnourished relative to malnourished broods from sufficiently nourished mothers (Vijendravarma et al., 2010). While the production of larger offspring in resource-limited environments may at first appear counterintuitive, the advantage lies in the positive correlation to progeny stress resistance and fitness.

What specific strategies of simple maternal effects have evolved and how? Computational models suggest that variable environmental conditions play a large part in the evolution of maternal effects. Kuijper and Hoyle (2015) find that constant environments minimize maternal effects to reduce maternal cost while still attaining an optimal phenotype, whereas sudden environmental shifts generate much stronger effects that persist for many generations. Additionally, selection is favored when mothers can accurately transmit information about the future conditions of the offspring, consistent with previous models (Uller, 2008; Kuijper and Hoyle, 2015). In temporally fluctuating environments, two predominant theories of maternal effects include deterministic effects and randomizing effects, which, respectively, refer to intentional, targeted investment and an increase in phenotypic variance among offspring. Though randomizing effects have been demonstrated in experimental evolution with *Neurospora crassa* (Graham et al., 2014), other experimental work has failed to generate similar findings (Dey et al., 2016). Recent theoretical work from Proulx and Teotonio (2017) indicates that natural selection is far more likely to favor the evolution of deterministic maternal effects as opposed to randomization; furthermore, randomizing effects are likely only to evolve if there is a total lack of predictability of a future conditions coupled with an environmental change of very narrowly defined parameters. Simple maternal effects constitute a diverse means of provisioning offspring with appropriate materials for survival, justifying further investigation from an evolutionary perspective.

## Terminal Investment

When an organism is challenged with a severe stressor and faces mortality as a result, an interesting investment phenotype may occur, in which resources are reallocated to favor reproductive success to the substantial detriment of somatic maintenance and organism survival. This is termed terminal investment and was originally used to describe the age-related rise in reproductive investment at the end of life, a topic which is still greatly debated (Clutton-Brock, 1984). Currently, the terminal investment hypothesis has been applied to a broad range of phenomena, from cellular to behavioral responses, generating some ambiguity in the exact characteristics required. Our discussion will be limited to only those cases of costly cellular investment that result from a high probability of relatively immediate mortality, jeopardizing future survival and reproduction of the parent. There are generally two forms of terminal investment that fit these criteria: in the first, the organism exposed to a stressor upregulates gamete production (and therefore reproductive capability); in the second, the overall quality of the offspring is improved as an extreme form of simple maternal effects (Duffield et al., 2017). Though the first strategy does not necessarily prepare offspring for the prevailing conditions, it does attempt to maximize the number of progeny that will survive to reproduce.

Examples of progeny number investment are abundant in nature, especially in response to infection. Brood size increases have been documented widely upon diverse pathogen exposure, extending to studies in snails, birds, and deer mice (Schwanz, 2008). Of particular contemporary relevance is the reproductive response demonstrated by frogs infected with the deadly chytrid fungal pathogen, *Batrachochytrium dendrobatidis* (Bd), which has devastated global amphibian populations for two decades (Fisher et al., 2009). Several studies have shown this infection to elicit upregulated gametogenesis in different species. Increases in gamete production and shifts in staging to favor higher concentrations of mature germ cells has been observed in both oogenesis and spermatogenesis, suggesting that this is not a sex-specific response (Chatfield et al., 2013; Brannelly et al., 2016). This is thought to have serious ramifications for the evolution of pathogen resistance, as terminal investment does not require that animals survive infection prior to reproducing, which would normally provide positive selection for Bd resistance; rather, terminal investment strategies only ensure population persistence (Brannelly et al., 2016). Despite this evidence, more thorough research is needed to ascertain whether these effects are adaptive on the part of the host or simply a side-effect of pathogenicity.

In the second case, terminal investment results in a general improvement to offspring quality by causing significant allocation of cytosolic resources to the individual gamete or embryo. In wild tsetse flies, smaller mass mothers, and perhaps more stressed mothers (as estimated by greater wing fray), may invest proportionally more body fat in their offspring, decreasing their own likelihood of survival (Hargrove et al., 2018). However, few examples of this phenomenon have been recorded, as most published studies report stress-dependent decreases in gamete quality and viability (El-Tarabany, 2016; Prasad et al., 2016). The possible ecological significance and paucity of investigation into this form of cytosolic terminal investment necessitates further research.

Likewise, very little mechanistic data exist to explain how terminal investment is elicited in response to various stressors and how it translates to life-history strategies that favor the survival of offspring over that of the parent. A comprehensive review of terminal investment from Duffield et al. (2017) posits an investment model based on dynamic thresholds for cues. In this model, terminal investment cues, rather than merely meeting an established threshold sufficient for the induction of the response, are integrated by the organism in the context of multiple other intrinsic (e.g., age, nutrition) and extrinsic factors (e.g., pathogen exposure) which increase or decrease their salience. Continuing to dissect terminal investment pathways may help elucidate how combinatorial factors produce this response. In terms of evolution, at least the cytosolic form of terminal investment appears to share similarities with simple maternal effects; it thus seems possible that this strategy evolved in response to severe regularly encountered stressors over prolonged periods of time.

Of the categories that we have explored, the terminal investment hypothesis, particularly as it pertains to gamete quality, is the least well-documented form of reproductive stress response. Continuing to develop our understanding of how organisms respond and integrate diverse genetic and environmental signals to maximize reproductive fitness has serious implications for evolutionary developmental biology, ecology, and even conservation, necessitating continued study and research of terminal investment at the cellular and molecular level.

## Concluding Remarks

**FIGURE 1:**
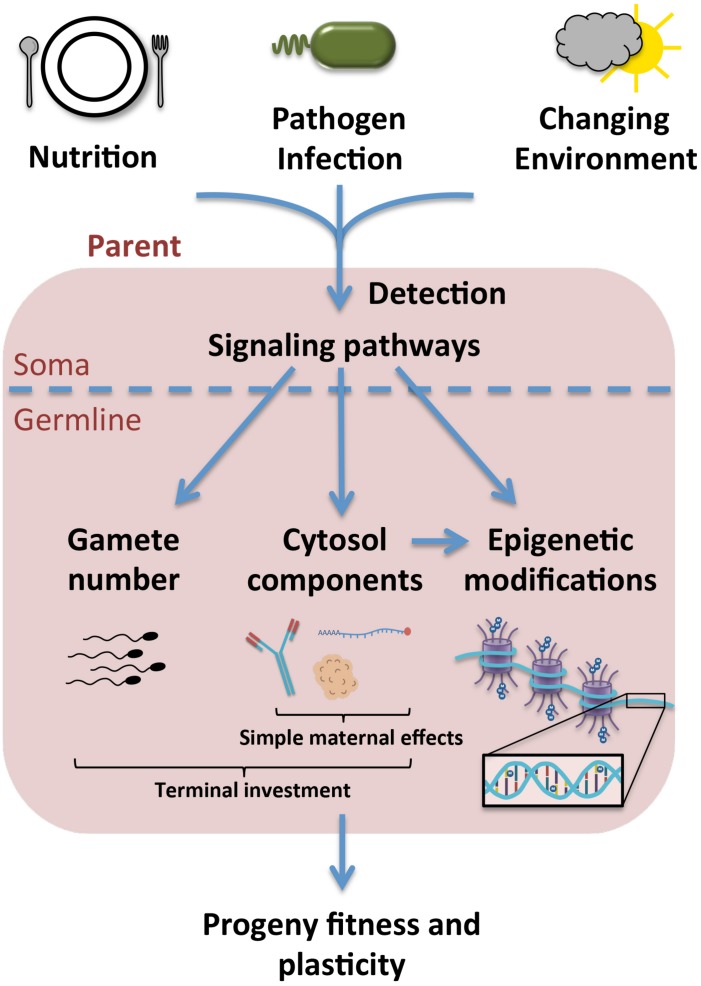
Upon exposure to stress, environmental signals are likely sensed in an organism’s soma and subsequently integrated into signals transmitted to the germline. This may result in the specific inclusion of certain macromolecules into the cytosol of the gamete or embryo, as in the case of simple maternal effects and some forms of terminal investment, or in the upregulation of gamete production as a means of terminal investment. More specific modifications may also be made to DNA or histones in the germline to regulate gene expression in the progeny, which can occur in the parental germline or as a result of the cytosolic environment experienced by the offspring during development. Ultimately these effects result in variations to progeny fitness and plasticity in a given environment.

An emerging understanding of the impacts of stress on reproduction and progeny fitness (Figure 1) has implications for numerous fields, including developmental and evolutionary biology and population genetics. Though epigenetics, simple maternal effects, and terminal investment were discussed here separately, it is important to note that these are not necessarily discrete categories. Aside from the similarities already noted between simple maternal effects and terminal investment, macromolecular cytoplasmic loading can also influence epigenetic changes in progeny. An example of this overlap is the inheritance of piRNAs in *Drosophila*, which in addition to silencing transposons, also regulate histone modifications to piRNA clusters (Le Thomas et al., 2014; Shpiz et al., 2014).

Parental effects at first appear to evade conventional theories of evolution, which would normally prohibit the transmission of life-history memory from one generation to the next. The complexity of researching and constructing models that adequately take into account the variation and intricacies of these mechanisms is challenging. A broadly applicable perspective is suggested by Badyaev and Uller (2009), who state that parental effects may give rise to “short-term context-dependent effects” which, over time, may become key developmental features subject to genetic selection.

Altogether, both biotic and abiotic stressors play a crucial role in molding the phenotypic plasticity of not just the immediately exposed generation, but also of subsequent generations. Rather than limiting reproduction, organisms may integrate environmental information and stress sensation to predict their own future mortality, as well as the environment of their offspring. Given the far-reaching repercussions of epigenetic inheritance, simple maternal effects, and terminal investment, further research is needed to generate a more comprehensive understanding of how these parental effects are elicited and carried out at the cellular level in response to environmental stimuli.

## Author Contributions

Both authors have made a substantial, direct and intellectual contribution to the work, and approved it for publication.

## Conflict of Interest Statement

The authors declare that the research was conducted in the absence of any commercial or financial relationships that could be construed as a potential conflict of interest.
